# Effect of Crystallinity on the Properties of Polycaprolactone Nanoparticles Containing the Dual FLAP/mPEGS-1 Inhibitor BRP-187

**DOI:** 10.3390/polym13152557

**Published:** 2021-07-31

**Authors:** Antje Vollrath, Christian Kretzer, Baerbel Beringer-Siemers, Blerina Shkodra, Justyna A. Czaplewska, Damiano Bandelli, Steffi Stumpf, Stephanie Hoeppener, Christine Weber, Oliver Werz, Ulrich S. Schubert

**Affiliations:** 1 Laboratory of Organic Chemistry and Macromolecular Chemistry (IOMC), Friedrich Schiller University, Humboldtstraße 10, 07743 Jena, Germany; antje.vollrath@uni-jena.de (A.V.); baerbel.beringer-siemers@uni-jena.de (B.B.-S.); Blerina.Shkodra-pula@uni-jena.de (B.S.); justyna.czaplewska@uni-jena.de (J.A.C.); damianobandelli@gmail.com (D.B.); steffi-stumpf@uni-jena.de (S.S.); s.hoeppener@uni-jena.de (S.H.); christine.weber@uni-jena.de (C.W.); 2 Jena Center for Soft Matter (JCSM), Friedrich Schiller University, Philosophenweg 7, 07743 Jena, Germany; oliver.werz@uni-jena.de; 3 Department of Pharmaceutical/Medicinal Chemistry, Institute of Pharmacy, Friedrich Schiller University, Philosophenweg 14, 07743 Jena, Germany; christian.kretzer@uni-jena.de

**Keywords:** polycaprolactone (PCL), polyesters, hydrophobic-hydrophilic balance (HHB), nanoparticle formulation, nanoparticle crystallinity, FLAP antagonist, BRP-187

## Abstract

Seven polycaprolactones (PCL) with constant hydrophobicity but a varying degree of crystallinity prepared from the constitutional isomers ε-caprolactone (εCL) and δ-caprolactone (δCL) were utilized to formulate nanoparticles (NPs). The aim was to investigate the effect of the crystallinity of the bulk polymers on the enzymatic degradation of the particles. Furthermore, their efficiency to encapsulate the hydrophobic anti-inflammatory drug BRP-187 and the final in vitro performance of the resulting NPs were evaluated. Initially, high-throughput nanoprecipitation was employed for the εCL and δCL homopolymers to screen and establish important formulation parameters (organic solvent, polymer and surfactant concentration). Next, BRP-187-loaded PCL nanoparticles were prepared by batch nanoprecipitation and characterized using dynamic light scattering, scanning electron microscopy and UV-Vis spectroscopy to determine and to compare particle size, polydispersity, zeta potential, drug loading as well as the apparent enzymatic degradation as a function of the copolymer composition. Ultimately, NPs were examined for their potency in vitro in human polymorphonuclear leukocytes to inhibit the BRP-187 target 5-lipoxygenase-activating protein (FLAP). It was evident by Tukey’s multi-comparison test that the degree of crystallinity of copolymers directly influenced their apparent enzymatic degradation and consequently their efficiency to inhibit the drug target.

## 1. Introduction

With the first clinical approval of a polymer-based nano-drug in 1995 [1], interest in developing polymers as nanocarriers of (bio)pharmaceutical drugs has been steadily growing [2,3]. Due to their favorable characteristics, e.g., adjustable physical and mechanical properties, it is not surprising that polymer-based nanomaterials are now established in many areas of bionanotechnology. Polymers are widely used in delivery systems for therapeutics, in matrices for tissue engineering and, among others, in polymer-based composites for biomedical purposes [4,5,6].

The main criteria in designing polymers for therapeutic use have been based mainly on the biocompatibility and the biodegradability of the polymer backbone as well as the suitability of the polymer to be processed into a stable pharmaceutical formulation [7]. However, there are other parameters of equal importance to be considered to optimize a polymer for its application as a delivery vehicle. In fact, parameters such as molar mass, functional end-groups, hydrophobic-hydrophilic balance (HHB), melting temperature (T_m_) and crystallinity strongly influence the drug loading and the drug release kinetics from the polymer matrix [8]. 

Independent investigations of the influence of the polymer crystallinity on the resulting particle characteristics (e.g., particle formation and degradation) while keeping the key properties of the system constant are rare or only provide partial conclusions typically due to influences of a third variable [9]. In particular, alterations of HHB are frequently accompanied by changes in crystallinity [10]. It is hence currently not fully understood if degradation or general performance of hydrophobic pharmapolymers in aqueous media are, in fact, strongly influenced by polymer crystallinity or if the hydrophobicity is the dominating factor.

Today, the most commonly used polymers for biomedical applications are polyesters, such as polylactide (PLA), poly(lactide-*co*-glycolide) (PLGA) and polycaprolactone (PCL) [11]. They are easy to access and offer a range of interesting advantages; i.e., (i) a complete hydrolytic and/or enzymatic biodegradation, (ii) a facile and controlled synthesis to obtain defined molar masses, (iii) various modification possibilities of the polymer structure, and (iv) commercial availability [12]. The advantages of PCL compared to other aliphatic polyesters include interesting thermal properties, higher durability and manufacturability, and a good compatibility with other polymers [13,14]. Thus, PCL represents a promising candidate to design materials with tailor-made properties [13,15,16]. Bandelli et al. recently demonstrated that copolymerization of the constitutional isomers ε-caprolactone (εCL) and δ-caprolactone (δCL) with a varying ratio of εCL and δCL can generate a library of five copolyesters featuring a constant HHB and similar molar masses in the range of 7 to 10 kDa, but the copolymers showed a varying crystallinity [17]. They are hence suitable materials to study the sole influence of crystallinity on the particle properties and performance. In this study, we utilized this library of poly(εCL-*ran*-δCL) to formulate drug-loaded nanoparticles (NPs). The aim was to investigate, firstly, whether such polymers provide suitable properties (particle size and polydispersity) to form an NP-based drug delivery system, and secondly, to study the effect of the crystallinity of the bulk polymers on the enzymatic degradation and the in vitro performance of the resulting NPs. 

The anti-inflammatory drug BRP-187 (4-(4-chlorophenyl)-5-[4-(quinoline-2-ylmethoxy)phenyl] isoxazol-3-carboxylic acid) is a dual inhibitor of the 5-lipoxygenase-activating protein (FLAP) and microsomal prostaglandin E2 synthase-1 (mPGES-1), which are crucial proteins within arachidonic acid (AA) metabolism. Inhibition of mPGES-1 and FLAP prevents the biosynthesis of pro-inflammatory prostaglandin (PG)E_2_ and leukotrienes (LTs), respectively [18]. Several in vitro and in vivo studies with inhibitors of FLAP and/or mPGES-1 have demonstrated their efficient anti-inflammatory activity while exhibiting fewer adverse effects compared to the conventional non-steroidal anti-inflammatory drugs (NSAIDs) [19,20]. These observations suggest that dual inhibition of FLAP and/or mPGES-1, rather than blocking cyclooxygenase-1 or -2 pathways, might be a better strategy for intervention with inflammation. However, BRP-187 is a fatty acid-like molecule with poor water solubility and a strong tendency to bind plasma proteins [18]. Molecules exhibiting such properties typically cause challenges in reaching a sufficient bioavailability in vivo and require technological solutions to improve their pharmacokinetic drawbacks. We have previously demonstrated that encapsulating BRP-187 into PLGA NPs and acetalated dextran NPs enhanced its enzyme inhibition efficacy in vitro [21].

In the present study, we initially performed a high-throughput (HT) nanoprecipitation approach for the homopolymers PεCL and PδCL to screen a range of polymer and surfactant concentrations for the preparation of empty (unloaded) PCL particles. Once optimal formulation conditions were established, and drug-loaded NPs were prepared by batch nanoprecipitation of the PεCL and PδCL homopolymers as well as of the poly(εCL-*ran*-δCL) copolymers with BRP-187. PCL NPs with and without BRP-187 were characterized for their critical quality attributes, namely their particle size, polydispersity index (PDI), surface charge and drug loading. Other particle properties i.e., nanodispersion stability and NP degradation behavior were also investigated. Ultimately, BRP-187-NPs were studied in vitro in human polymorphonuclear leukocytes (PMNL) for their efficiency to inhibit the drug target FLAP, in comparison to the free BRP-187.

## 2. Methods

### 2.1. Materials

The PCL homopolymers and P(εCL-*ran*-δCL) copolymers were synthesized as previously reported [17]. Key characterization data are listed in Table S1 in the Supplementary Information (SI). For further details on their synthesis, the reader is referred to literature reports [17]. Polyvinylalcohol (PVA) (Mowiol 4-88), tetrahydrofuran (THF), dimethylsulfoxide (DMSO) and lipase from the yeast *Candida rugosa* were purchased from Sigma-Aldrich (Germany). BRP-187 was synthesized according to a published protocol [18]. Further materials are described in the specific experimental sections.

### 2.2. Automated High-Throughput Nanoprecipitation

Automated high-throughput nanoprecipitation was performed in a 96-well plate (Greiner Bio-One GmbH, Frickenhausen, Germany) utilizing a FasTrans liquid handling robot (Analytik Jena GmbH, Jena, Germany). Starting with a polymer stock solution of 10 mg mL^−1^ in THF, a dilution series with varying concentrations (0.25, 0.5, 1, 2, 3, 4, 5, 6, 7, 8, 9 and 10 mg mL^−1^) was prepared. The polymer solutions (40 µL) were then automatically pipetted into 200 µL of either purified water (GenPure ultrapure water system, Thermo Scientific, Waltham, MA, USA) or PVA surfactant-containing aqueous solutions with a concentration of 0.25%, 0.5% or 1.0% (*w/v*). The resulting NP dispersions were mixed by pipetting up and down three times and then left for two hours for solvent evaporation. Each formulation was prepared twice. After solvent evaporation, the samples were diluted with pure water (1:2 ratio for a polymer concentration up to 4 mg ml^−1^ and 1:10 ratio for all NPs prepared with a polymer concentration above 4 mg mL^−1^) and investigated via dynamic light scattering (as described in Section 2.4) [22]. 

### 2.3. Batch Nanoprecipitation

Polymer solutions with 5 mg mL^−1^ or 2.5 mg mL^−1^ were prepared in THF via batch nanoprecipitation. For the drug-loaded particles, 10 mg mL^−1^ of BRP-187 dissolved in DMSO were mixed with the polymer solution prior to formulation, which corresponded to 3% (w/w) of the drug to polymer mass. The drug stock solution was sonicated in an ultrasound water bath for 15 min at room temperature to ensure good dissolution. Particle formulation was carried out by injecting the polymer/drug solution into an aqueous phase containing 0.3% (*w/v*) PVA using a syringe pump (Aladdin AL1000-220, World Precision Instruments, Berlin, Germany) with a flow rate of 2 mL min^−1^ while stirring at 800 rpm. The solvent/non-solvent ratio was set to 1:8. The resulting particle suspensions were stirred for 24 h at room temperature for solvent evaporation and then centrifuged at 12.851 *x g* for 60 min at 20 °C using a Rotina 380 R centrifuge (Hettich Lab Technology, Tuttlingen, Germany). The supernatant was removed, and the NPs were redispersed in 2.5 mL pure water, vortexed and sonicated in an ultrasonic water bath for 30 min. The NPs were stored overnight at 4 °C and lyophilized in aliquots of 200 µL. After lyophilization, the mass of the NPs was determined using a precise analytical balance (MYA 11.4Y, Radwag Waagen, Hilden, Germany). The yield was calculated as follows: (mass of NPs recovered – mass of found PVA)/(mass of polymer + mass of drug) in the formulation × 100. To check reproducibility, five individual batches of the drug-loaded PCL particles were prepared and analyzed individually. The data provided represent the average values and the standard deviation of these five batches.

### 2.4. Dynamic Light Scattering (DLS) and Electrophoretic Light Scattering (ELS)

DLS measurements were performed utilizing a Nano ZS (Malvern Panalytical, Malvern, United Kingdom) with a laser wavelength of λ = 633 nm with non-invasive back-scatter (NIBS) technology [22]. The particle size is reported as the hydrodynamic diameter (d_H_). The particle size distribution (PDI) was measured using pure water as a dispersant with a refractive index RI of 1330 and a viscosity of 0.8872 cP at 25 °C. Samples obtained from the automated HT-nanoprecipitation were measured at 25 °C in a micro cuvette (Brand GmbH, Wertheim, Germany) without any filtering step with the following settings: measurements of each sample were repeated three times for 10 sec at 25 °C. The samples obtained from batch nanoprecipitation were measured at a dilution of 1:10 up to 1:100 utilizing the following settings: five repeated measurements, each with five runs of 30 s. The zeta-potential of the lyophilized NPs was investigated by ELS using the same instrument at 25 °C with three repeated measurements. 

The apparent degradation behavior of the NPs was analyzed by DLS by monitoring changes in the mean count rate at fixed measurement settings: measuring position at 4.65, attenuator factor 7 at 37 °C [21]. Before investigating, NPs were mixed with the enzyme solution (a lipase from Candida rugosa) in a 1:4 mass ratio of polymer to enzyme and incubated at 37 °C for pre-determined timepoints. 

### 2.5. UV-Vis Spectroscopy Measurements

UV-Vis spectroscopy measurements were performed with the Infinite M200 Pro plate reader (Tecan Group, Männedorf, Switzerland). For determination of the encapsulation efficiency (EE) and the loading capacity (LC) of the BRP-187 in the PCL particles, lyophilized NPs were dissolved in DMSO, and the solutions were investigated in a flat-transparent 96-well quartz plate (Hellma, Jena, Germany) at λ = 316 nm with 3 × 3 multiple reads per well and a 2000 µm well border. A calibration curve of BRP-187 was obtained for each batch in the concentration range of 1.2 to 312.5 µg mL^−1^ with R^2^ = 0.9997. The LC was calculated as follows: LC = (mass of drug recovered)/(mass of particle recovered) x 100. The EE was calculated as follows: EE = LC found/(mass of drug used) × 100. The determination of PVA in the NPs (%, *w/w*) was performed according to the published protocol [23]. 

### 2.6. Scanning Electron Microscopy (SEM)

A Sigma VP Field Emission Scanning Electron Microscope (Carl-Zeiss, Jena, Germany) equipped with an InLens detector with an accelerating voltage of 6 kV was used for electron microscopy imaging. Before the measurement, the samples were coated with a thin layer of platinum (4 nm) via sputter coating (CCU-010 HV, Safematic, Zizers, Switzerland). 

### 2.7. Cell Isolation

The leukocytes isolation was performed according to a published protocol [21]. Leukocyte concentrates were prepared from peripheral blood obtained from healthy human adult donors that received no anti-inflammatory treatment for the last ten days (Institute of Transfusion Medicine, University Hospital Jena). The approval for the protocol was given by the ethical committee of the University Hospital Jena, and all methods were performed in accordance with the relevant guidelines and regulations. To isolate PMNL, the leukocyte concentrates were mixed with dextran (*Leuconostoc* spp. MW ~40,000, Sigma Aldrich, Taufkirchen, Germany) for sedimentation of erythrocytes and the supernatant was centrifuged on lymphocyte separation medium (Histopaque^®^-1077, Sigma Aldrich, Taufkirchen, Germany). Contaminating erythrocytes in the pelleted neutrophils were removed by hypotonic lysis (water). PMNL were then washed twice in ice-cold phosphate-buffered saline (PBS) and finally resuspended in PBS plus 0.1% of glucose and 1 mM CaCl_2_.

### 2.8. Determination of FLAP-Dependent 5-LO Product Formation in PMNL

The evaluation of the effects on FLAP was performed according to our established protocol [21]. We assessed FLAP-dependent 5-LO product formation in human PMNL, cells (5 × 10^6^ mL^−1^) were pre-incubated with BRP-187 or NPs for indicated timepoints at 37 °C. The cells were stimulated with 2.5 µM Ca^2 +^ -ionophore A23187 (Cayman, Ann Arbor, USA) for 10 min, and the incubation was stopped with 1 mL ice-cold methanol containing 200 ng mL^−1^ PGB_1_ as an internal standard. Samples were subjected to solid phase extraction, and the formed lipid mediators (leukotriene B_4_ (LTB_4_), trans-isomers of LTB_4_, 5-hydroxyeicosatetraenoic acid (5-HETE)) were separated and analyzed by reverse-phase high-performance liquid chromatography (RP-HPLC) as previously described [24]. Statistical analysis was performed with log-transformed values to obtain Gaussian-distributed data sets. Experiments were analyzed via one-way ANOVA and Tukey’s multicomparison test with GraphPad Prism 9.1.2 (GraphPad, La Jolla, CA, USA).

### 2.9. Cell Viability

Freshly isolated PMNL were incubated with a control sample with 0.1% DMSO, BRP-187 (10 µM) or NPs containing the respective amount of BRP-187 (10 µM) at 37 °C in PBS containing 0.1% of glucose. After 5 h the cell suspension was subjected to a Vi-CELL XR cell counter (Beckman Coulter, Lahntal, Germany), for determination of cell viability by trypan blue staining.

## 3. Results and Discussion

**Figure 1:**
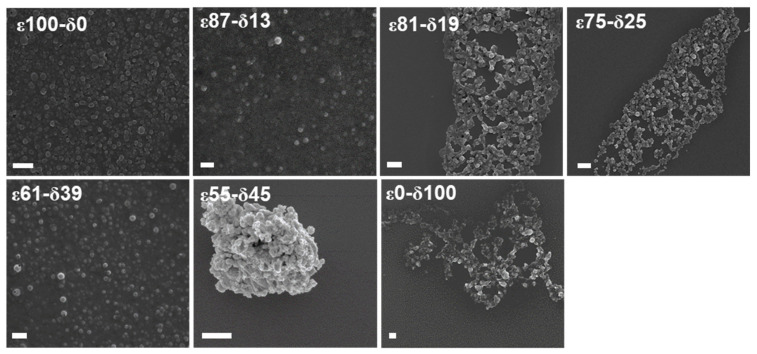
SEM micrographs of PCL[BRP-187] particles consisting of the homo- or copolymers with a varying composition. Scale bar = 1 µm.

In our previous study, five poly(εCL-*ran*-δCL) copolymers, herein named ε87-δ13, ε81-δ19, ε75-δ25, ε61-δ39 and ε45-δ55, and the two respective homopolymers PεCL and P*δ*CL, herein referred to as ε100-δ0 and ε0-δ100, were synthesized exhibiting a constant HHB [17]. It was demonstrated that the HHB of the bulk polymers correlated with the HHB of the corresponding NPs when particles were prepared in THF using a polymer concentration of 1 mg mL^−1^ [17]. In the present study, the particle formation of the ε100-δ0 and ε0-δ100 was investigated over a wider range of polymer concentrations in THF ranging from 0.25 to 10 mg mL^-1^ using an automated pipetting robot that was adapted for the HT-nanoprecipitation [25]. Particles were formulated without surfactant as well as with PVA of different concentrations (0.25 to 1% (*w/v*)). Previous studies revealed that PVA of less than 0.5% (*w/v*) generated stable drug-loaded PLGA NPs, and it could be demonstrated that even concentrations of up to 5% (*w/v*) were generally non-toxic in vitro [26]. ε100-δ0 and ε0-δ100 homopolymers both formed NPs up to the highest tested polymer concentration of 10 mg mL^−1^ when PVA was used as a surfactant (SI, Figure S1). Even the lowest tested PVA concentration of 0.25% (*w/v*) was sufficient to obtain stable particle dispersions and ε100-δ0 NPs with a size of 150 to 300 nm and ε0-δ100 NPs with a particle size of 120 to 280 nm with PDI < 0.3. However, ε0-δ100 NPs prepared without surfactant failed to produce stable NP dispersions above concentrations of 0.5 mg mL^−1^ as indicated by a strong aggregation of the particles. This is not surprising since ε0-δ100 is above its glass transition temperature at room temperature, which could disturb the particle formation in the absence of a stabilizer. It is well-known that several factors influence the final NP properties, including the polymer concentration, the solvent used to dissolve the polymer and the type and the concentration of the surfactant [26,27,28]. THF was demonstrated to be a suitable solvent in the HT-screening, resulting in stable particle formation within a broad polymer concentration range when PVA was used as a surfactant. Hence, it was selected as solvent for the subsequently performed BRP-187 encapsulation experiments. All other formulation parameters for the preparation of PCL[BRP-187] NP were adapted from our previous study that described the encapsulation of BRP-187 into PLGA NPs [21]. The first batch nanoprecipitation with the drug and a polymer concentration of 5 mg mL^−1^ in THF yielded large particles with a diameter (d_H_) of 400 to 600 nm with high LC values (SI, Table S2). However, the particles revealed significant aggregation after centrifugation and lyophilization, as indicated by the higher PDI values of 0.3 to 0.6. Hence, the initial polymer concentration was reduced to 2.5 mg mL^−1^ to optimize the dispersion stability and to decrease the particle size [28]. Particles within a size range of 200 to 260 nm and PDI values below 0.3 were obtained for all PCLs using a polymer concentration of 2.5 mg mL^−1^ (Table 1, SI Table S4). It was further observed that empty NPs were approximately 30 to 50 nm smaller compared to the BRP-187-loaded NPs (SI, Table S3). The particle size of the empty NPs increased by approximately 40 to 80 nm when NPs were lyophilized and subsequently reconstituted in water (SI, Table S3). Similar tendencies were also observed for the PCL[BRP-187] NPs, although here the difference in size was on average only about 30 to 50 nm (Table 1), presumably caused by the strong affinity of the hydrophobic drug with the polymer matrix [29]. The particles were also investigated via SEM (Figure 1), which revealed individual or clustered particle populations within the particle size range as indicated by DLS measurements.

The average LC of the PCL[BRP-187] NPs was between 1.4 and 1.9% for ε100-δ0 and the poly(εCL-*ran*-δCL) copolymers (Table 1) and similar to the LC values of PLGA NPs encapsulating the same drug [21]. The only exception was the ε0-δ100 homopolymer with an LC of 3.2%, probably due to its almost liquified state at room temperature. This resulted in a viscous dispersion with emulsion-like properties in which the drug was apparently entrapped during the purification process.

**Figure 2:**
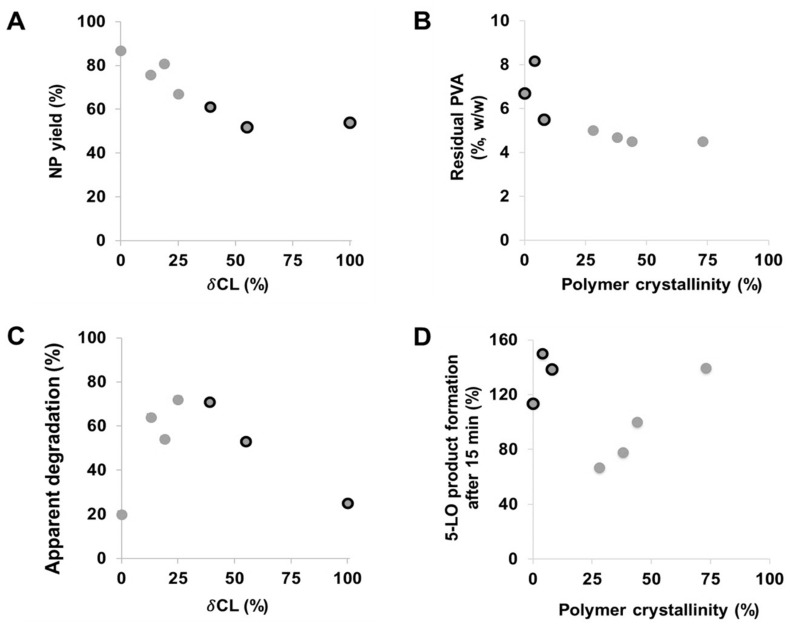
Influence of the δCL fraction on the yield of drug-loaded PCL NPs (**A**), influence of the polymer crystallinity on the residual PVA content of drug-loaded PCL NPs (**B**), apparent degradation represented by the normalized relative count rate (%) after 20 h plotted against the δCL fraction of the copolymers (**C**) and influence of polymer crystallinity on the efficiency of drug-loaded PCL NPs to inhibit 5-LO product formation (**D**). Black-circled data points represent PCL polymers with a degree of crystallinity below 10% and a T_g_ < 37 °C.

In general, the yield of both empty and drug-loaded PCL NPs decreased with increasing molar fraction of *δ*CL (Figure 2A). In other words, NP yield increased with the degree of crystallinity of the polyester materials. Amorphous materials are frequently utilized as excipients in pharmaceutical formulations since they are known to increase the dissolution rate of insoluble drugs and to enhance their bioavailability [30]. However, their major disadvantage is seen in the fact that they exhibit high energy states at a molecular level and thus are prone to physical instabilities. In particular, such tendencies were observed with the NPs of the amorphous PδCL homopolymer, which displayed a higher polydispersity and the lowest yield. In technical terms, the low yield of the copolymers with a higher fraction of δCL could have resulted from their near-molten state at room temperature causing them to sediment at a lower rate due to their lower density. Thus, after 60 min of centrifugation, a lower amount of the NPs was recovered. 

Furthermore, it was observed that the residual amount of PVA in the drug-loaded NPs was higher compared to the empty NPs for all PCL copolymers (Table 1 and SI, Table S3). As mentioned before, such differences are typically a result of strong drug–polymer interactions [31], and in this case, the interactions of the BRP-187 with the chains of PVA polymer. Moreover, the residual PVA content was noticeably higher for less crystalline copolymers with a higher δCL fraction and highest for the particles consisting of the PδCL homopolymer (Figure 2B). Apparently, the surfactant molecules tended to stick to the surface or were even incorporated into the particles formed from amorphous polyesters that are above their glass transition temperature during formulation. As soon as the materials were semicrystalline and below T_m_, the degree of crystallinity did not influence the amount of residual PVA anymore. Besides providing dispersion stability, surfactants also influence the degradation rate of NPs since they adsorb at the surface of the particles forming a layer that protects from enzymatic hydrolysis to some degree [32]. Additional characterization experiments of the PCL[BRP-187] NPs were performed to investigate the degradation kinetics as well as the biological evaluation of the NP efficiency to inhibit the drug targets in vitro.

### 3.1. Degradation Studies

**Figure 3:**
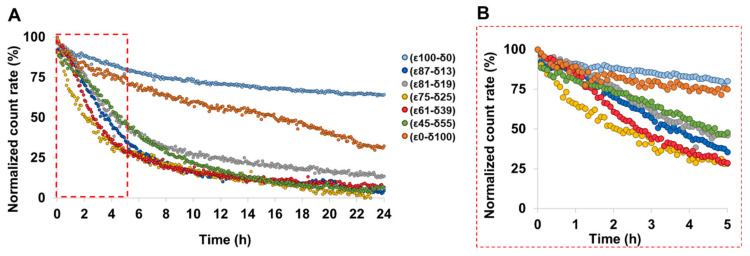
Normalized count rate of BRP-187-loaded PCL NPs incubated with *Candida rugosa* as measured by DLS for 24 h (**A**). (**B**) depicts a zoomed-in area into the data until 5 h.

Among the aliphatic polyesters that are most commonly investigated for drug delivery applications, PCL has a superior thermal stability, with a decomposition temperature of 100 °C higher above that of the typical PLA- and PGA-based polymers [15]. Due to its high durability, PεCL has found a wide range of applications mainly for implantable medical devices [33,34], in which degradation occurs over two to four years [13]. However, to tailor their application for drug delivery purposes, faster degradation kinetics of the P*ε*CL are desirable and can be achieved by copolymerization of εCL with its isomer δCL [9]. Introducing δCL repeating units to the PεCL polymer decreases its degree of crystallinity [17], and as such, it increases its rate of degradation as confirmed by investigations of films [35]. Figure 3 shows the enzymatic degradation of the PCL[BRP-187] particles incubated for 24 h at 37 °C as monitored by DLS. The apparent NPs degradation was inferred by monitoring changes in the sample concentration over time, as indicated by the count rate on the DLS under constant measurement settings [21]. In agreement with literature reports regarding film degradation, Figure 3 reveals that the degradation of the most crystalline ε100-δ0 was the slowest in the nanoparticulate state. ε100-δ0 is predominantly a semicrystalline material with a melting point considerably higher than the experimental temperature of 37 °C. It was noticed that except for the ε0-δ100 homopolymer, which degraded only about 25% after 24 h, the NP degradation rate generally increased with the amount of the δCL (Figure 3A). This was expected since the long-range order and the compact structure of crystalline materials requires higher levels of energy for degradation compared to the less organized molecular arrangement of amorphous materials [29,36].

This observation is further confirmed by other studies that have also demonstrated that the amorphous regions within bulk and/or films of PεCL polymer degraded faster compared to the crystalline regions [35,37,38]. Another study with similar observations argued that polyesters with higher crystallinity exhibit a slower degradation because in a densely packed crystal, it is more difficult for the enzymes to reach the cleavable bonds [39]. In general, our results revealed that all copolyester NPs featured an apparent degradability above 50% within 5 h (Figure 3B). A faster initial degradation was particularly observed for the ε75-δ25 and ε61-δ39 copolymers since they exhibit melting points (42 °C and 24 °C, respectively [17]) that are closer to the experimental temperature of 37 °C, which was chosen to simulate the conditions of the human body (Figure 2C and Figure 3B) [8]. 

### 3.2. In vitro Performance of NPs

**Figure 4:**
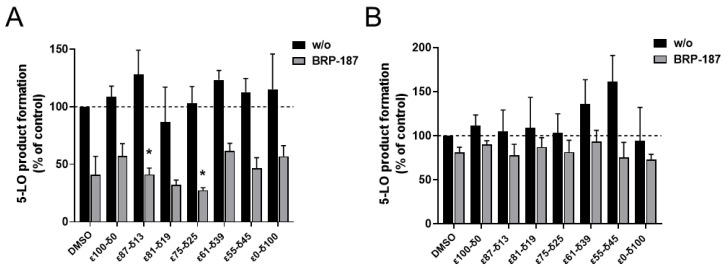
Measurement of 5-LO product formation as an indicator for the inhibition of the drug target FLAP by BRP-187 [36]. PMNL preincubated with DMSO, BRP-187 (0.3 µM), empty PCL particles (labeled as w/o) or PCL particles with BRP-187 (labeled with BRP-187; 0.3 µM respective BRP-187) for 15 min (**A**) or 5 h (**B**) at 37 °C. Values are given as 5-LO products as a percentage of control (DMSO) (n = 3). Statistical analysis was performed via one-way ANOVA and Tukeys multi comparison test with logarithmic trans-formed data (* *p* < 0.05).

Although clear influences of the polymer crystallinity and physical state on NP formulation and enzymatic degradation were found, other effects might come into play in the more complex environment of a cell. The PCL[BRP-187] particles were hence tested in human PMNL for bioactivity. PMNL are the most abundant leukocytes in the blood and are a major source for FLAP-dependent 5-LO product biosynthesis, thus they are suitable cells for evaluation of various anti-LT agents. Note that FLAP as helper-protein of 5-LO has no enzyme activity that can be experimentally assessed, but instead assists 5-LO in LT formation by facilitating the access towards the substrate for the 5-LO enzyme. At first, the PCL[BRP-187] NPs were compared to the free drug for their influence on the cell viability of PMNL (cytotoxicity). No cytotoxic effects of the particles were found within a 5 h incubation as shown in the SI (Figure S2). These results were in agreement with previous studies that demonstrated PCL NPs to be biocompatible [40,41]. Considering their good biocompatibility, all PCL[BRP-187] particles as well as the free drug were studied for their efficiency to inhibit the drug target FLAP in PMNL and, thus, to prevent 5-LO product formation [42]. Therefore, a drug concentration of 0.3 µM was chosen for free and encapsulated BRP-187, which were investigated at different preincubation times (15 min, 1 h, 2 h and 5 h, respectively). As shown in Figure 4A, 5-LO product formation was clearly suppressed after 15 min of incubation with the PCL[BRP-187] particles to variant degrees, but essentially the particles performed as efficiently as the free drug. Apart from this, there was no significant difference between the different PCL[BRP-187] polymers at longer incubation time points (i.e., 1–5 h; Figure 4B and SI, Figure S3). More specifically, the NPs prepared with ε75-δ25 prevented the 5-LO product formation most after 15 min of incubation (Figure 4A). This observation also correlated with the fastest apparent degradation of the ε75-δ25 copolymer (Figure 3B), which might be promoted by its melting point of 42 °C, which is around the temperature of cell incubation (i.e., 37 °C). Karavelidis et al. reported that other polyesters with melting points around 37 °C exhibited a faster drug release [8]. It can be inferred that the rapid degradation of ε75-δ25 led to an accelerated release of the BRP-187, thereby considerably preventing the 5-LO product formation at early time points (Figure 2C,D). NPs formed from PCL with higher εCL fraction and hence higher T_m_, as well as a higher degree of crystallinity, were less effective. As shown in Figure 2D, the 5-LO product formation was almost linearly dependent on the polymer crystallinity if only the semicrystalline materials are considered. The better performance of the NPs with lower crystallinity could be explained by two effects based on two different release mechanisms. Firstly, less crystalline materials with a larger fraction of amorphous domains enable a faster diffusion of the drug through the polymer matrix without barriers formed by crystalline domains [11,43]. Secondly, if the drug release is promoted through polymer degradation, these amorphous domains would most likely be more accessible for enzymes catalyzing the polyester hydrolysis [11]. 

Based on the apparent enzymatic degradation of the PCL NPs, a burst release of the drug is conceivable considering the immediate decrease in the count rate of at least 5 to 10% of all PCL NPs (Figure 3B). The slightly higher efficiency of the NPs to inhibit 5-LO product formation in PMNL within 15 min supports this idea (Figure 4A). For the polymers with a degree of crystallinity below 10% and T_g_ < 37 °C, namely ε61-δ39, ε55-δ45 and ε00-δ100, inhibition of 5-LO product formation is less apparent after 15 min of incubation (Figure 2D, black-circled data points). This is presumably because these polymers are molten and more viscous at 37 °C. As a consequence, they could delay the release of the drug and therefore hamper the drug action in the cells. 

Furthermore, the coating effect of PVA might reduce the influence of the crystallinity of NPs or their intracellular drug release. It is reported that increasing amounts of residual surfactant decrease the cellular uptake of the NPs [44,45]. This could explain why the PCL copolymers with a higher fraction of δCL containing more residual PVA (Figure 2B) were less efficient to inhibit 5-LO product formation after 15 min of incubation (Figure 2C, Figure 4A) when compared to the PCL copolymers with a higher εCL fraction containing less residual PVA. However, no correlation was observed between suppression of 5-LO product formation and the PVA content in the formulation, showing that the trend cannot be generalized (SI, Figure S4).

## 4. Conclusions

A library of poly(εCL-*ran*-δCL) copolymers with a constant HHB but different degrees of crystallinity were used to encapsulate BRP-187 into polymer NPs. PCL[BRP-187] particles with a diameter of 200 to 300 nm were successfully produced, whereby a comparable drug-loading was observed with LC between 1.4% and 1.9%, with the exception of the PδCL homopolymer, which revealed a higher LC. It was evident that the degree of crystallinity directly influenced the enzymatic degradation rate of the PCL copolymer, whereby the degradation increased with an increasing fraction of δCL repeating units. In addition, increasing the amount of δCL in the polymer increased the amount of residual surfactant in the NP formulation but decreased the final NP yield. The release of bioactive BRP-187 from the PCL NPs was demonstrated in vitro in PMNL by inhibiting FLAP-dependent 5-LO product formation, whereby the inhibition efficiency was dependent on the degree of crystallinity of the copolymers used for the particle formulation. The NPs of ε75-δ25 revealed the fastest degradation and inhibited the 5-LO product formation more than the other copolymers after 15 min of incubation in PMNL; longer preincubation times (1 to 5 h) reduced the potency. In conclusion, although all PCL copolymers were suited to produce NPs, the ε75-δ25 copolymer can be considered as a more promising candidate to be further investigated for both its physicochemical properties and its performance in more complex biological models. When designing superior materials for NP-mediated drug delivery, it hence seems promising to rely on polymers that are in a solid state of matter at 37 °C but feature a low degree of crystallinity. However, it is not yet clear if these observations can be applied to other systems. Thus, our future research will concentrate on the encapsulation of other anti-inflammatory drugs in the polymer library with constant HHB to determine if the effect of polymer crystallinity of the present PCL systems can be transferred to other actives. In addition, we are currently establishing similar libraries mimicking the HHB of PLA to understand if our findings can be generalized in the field of polyester-based drug carrier materials.

## Figures and Tables

**Table 1:** Overview of PCL[BRP-187] NP properties prepared in THF using a polymer concentration of 2.5 mg mL^−1^.

εCL/δCL (mol %)	T_m_ (°C)	X_c_^a^ (%)	d_H_^b^ (nm)	PDI ^b^	ZP ^c^ (mV)	d_H_^c^ (nm)	PDI ^c^	PVA % (*w/w*)	Yield ^d^ (%)	LC ^e^ (%)
ε100-δ0	69	73	229 ± 13	0.08 ± 0.02	−50 ± 1	268 ± 21	0.27 ± 0.09	4.5	87	1.5 ± 0.1
ε87-δ13	54	44	211 ± 5	0.08 ± 0.02	−38 ± 2	251 ± 13	0.30 ± 0.14	4.5	76	1.4 ± 0.5
ε81-δ19	52	38	218 ± 13	0.08 ± 0.02	−41 ± 1	267 ± 24	0.37 ± 0.27	4.7	81	1.4 ± 0.2
ε75-δ25	42	28	225 ± 13	0.16 ± 0.11	−34 ± 1	260 ± 23	0.42 ± 0.20	5.0	67	1.9 ± 0.6
ε61-δ39	24	4	209 ± 13	0.06 ± 0.12	−40 ± 1	223 ± 16	0.16 ± 0.10	8.2	61	1.7 ± 0.1
ε45-δ55	/ ^*^	0	200 ± 13	0.10 ± 0.12	−32 ± 1	237 ± 61	0.18 ± 0.08	6.7	52	1.4 ± 0.2
ε0-δ100	/ ^*^	8	259 ± 32	0.28 ± 0.14	−45 ± 2	262 ± 20	0.26 ± 0.26	5.5	54	3.2 ± 1.2

d_H_ represents the intensity-weighted distribution (n ≥ 4 batches) and zeta-potential (ZP) (n = 3 ELS measurements) * Amorphous or near amorphous polymers with glass transition temperature T_g_ below 37 °C [17]. ^a^ Bulk degree of crystallinity as determined by wide-angle X-ray scattering (WAXS) at room temperature. ^b^ NPs measured after purification. ^c^ NPs measured after lyophilization and subsequent resuspension in water. ^d^ Yield = (mass of NPs recovered – mass of found PVA)/(mass of polymer + mass of drug) in the formulation × 100. ^e^ Determined by UV-VIS spectroscopy at λ = 316 nm (n = 4) and calculated using LC = (mass of drug recovered)/(mass of particle recovered) × 100.

## Data Availability

The data presented in this study are available on request from the corresponding author.

## Supplementary Materials

The following are available online at https://www.mdpi.com/article/10.3390/polym13152557/s1, Figure S1: Hydrodynamic diameter (intensity-weighted distribution, circles) and PDI (bars) of the homopolymer ε100-δ0 and ε0-δ100 NPs over a range of PVA concentration used in the formulation, Figure S2: Cell viability measured with a Beckman ViCell XR cell counter by trypan blue staining. A total of 1 × 107 PMNL were diluted in PBS plus 0.1% of glucose and incubated with DMSO, BRP-187 (10 µM), empty PCL particles (labeled as w/o) or PCL particles with BRP-187 (labeled with BRP-187; respective amount to 10 µM BRP-187) for 5 h at 37 °C. Values are given as 5-LO products as a percentage of control (DMSO) (n = 3), Figure S3: Measurement of 5-LO product formation as indicator for the inhibition of the drug target 5-lipoxygenase-activating protein (FLAP) by BRP-187.[37] A total of 5 × 106 polymorphonuclear leukocytes (PMNL) diluted in PBS containing 0.1% glucose and 1mM CaCl_2_ were preincubated with DMSO, BRP-187 (0.3 µM), empty PCL particles (labeled as w/o) or PCL particles with BRP-187 (labeled as BRP-187; 0.3 µM respective BRP-187) for 1 h (A) and 2 h (B) at 37 °C and further stimulated with 2.5 µM A23187 for 10 min. The reaction was stopped with 1 mL ice-cold methanol containing 200 ng mL-1 PGB1 as internal standard. Lipid mediators were extracted via solid-phase extraction (SPE) and analyzed with HPLC. Values are given as 5-LO products (LTB4, its trans-isomers 4 and 5-HETE) as a percentage of control (DMSO) (n = 3), Figure S4: Influence of the residual PVA on the efficiency of drug-loaded PCL NPs on 5-LO inhibition. Black-circled data points represent PCL polymers with bulk degree of crystallinity below 10% and glass transition temperature Tg < 37 °C. Table S1: Molar mass and composition of the (co)polyesters. Details are described in a previous publication, Table S2: Properties of PCL[BRP-187] NPs formulated from THF utilizing polymer concentration of 5 mg mL^−1^ (n = 1 batch), Table S3: Particle properties of empty PCL NPs prepared in THF with c = 2.5 mg mL 1 (n = 2 batches) obtained by DLS and ELS measurements after purification and after lyophilization and subsequent resuspension (n = 2 for purified NPs, n = 1 for lyophilized NPs), Table S4: DLS intensity-weighted size distribution of PCL[BRP-187] NPs of one formulation round after purification, as well as after lyophilization and resuspension in water.
