# Supplementary material for: Effect of Crystallinity on the Properties of Polycaprolactone Nanoparticles Containing the Dual FLAP/mPEGS-1 Inhibitor BRP-187
Source: Polymers (Basel). 2021 Jul 31;13(15):2557. doi: 10.3390/polym13152557 (PMC8347491; doi:10.3390/polym13152557)
Supplement: Supplementary file 1 [file polymers-13-02557-s001.zip › polymers-1297780-supplementary.pdf]

# Supporting information

## Effect of crystallinity on the properties of polycaprolactone nanoparticles containing the dual FLAP/mPEGS-1 inhibitor BRP-187-187

Antje Vollrath [a, b], Christian Kretzer [c], Bärbel Beringer-Siemers [a], Blerina Shkodra [a, b], Justyna A. Czaplewska [a], Damiano Bandelli [a, b], Steffi Stumpf [a, b], Stephanie Hoeppener [a, b], Christine Weber [a, b], Oliver Werz [b, c], Ulrich S. Schubert [a, b] *

* Corresponding author

[a]Laboratory of Organic Chemistry and Macromolecular Chemistry (IOMC)
Friedrich Schiller University, Humboldtstraße 10, 07743 Jena, Germany

[b]Jena Center for Soft Matter (JCSM)
Friedrich Schiller University, Philosophenweg 7, 07743 Jena, Germany

[c]Department of Pharmaceutical/Medicinal Chemistry, Institute of Pharmacy
Friedrich Schiller University, Philosophenweg 14, 07743 Jena, Germany

**Table S1.** Molar mass and composition of the (co)polyesters. Details are described in a previous publication.[1]

| **εCL/ δCL[a] (mol %)** | **$M_{n, theo}$[b] (kg mol$^{-1}$)** | **$M_{n, NMR}$[a] (kg mol$^{-1}$)** | **$M_{n, SEC}$[c] (kg mol$^{-1}$)** | **$Đ_{SEC}$[c]** |
|---|---|---|---|---|
| 100/0 | 11 | 13 | 19 | 1.17 |
| 87/13 | 9 | 13 | 21 | 1.57 |
| 81/19 | 9 | 10 | 19 | 1.41 |
| 75/25 | 8 | 10 | 19 | 1.30 |
| 61/39 | 7 | 9 | 16 | 1.26 |
| 45/55 | 7 | 7 | 15 | 1.21 |
| 0/100 | 10 | 9 | 6 | 1.09 |

(a) Determined by $^1$H-NMR spectroscopy of the purified polymers.
(b) Molar mass expected from monomer conversions and the feed ratio.
(c) Determined by size exclusion chromatography (eluent $CHCl_3$, refractive index detection, polystyrene calibration).

## Supporting information

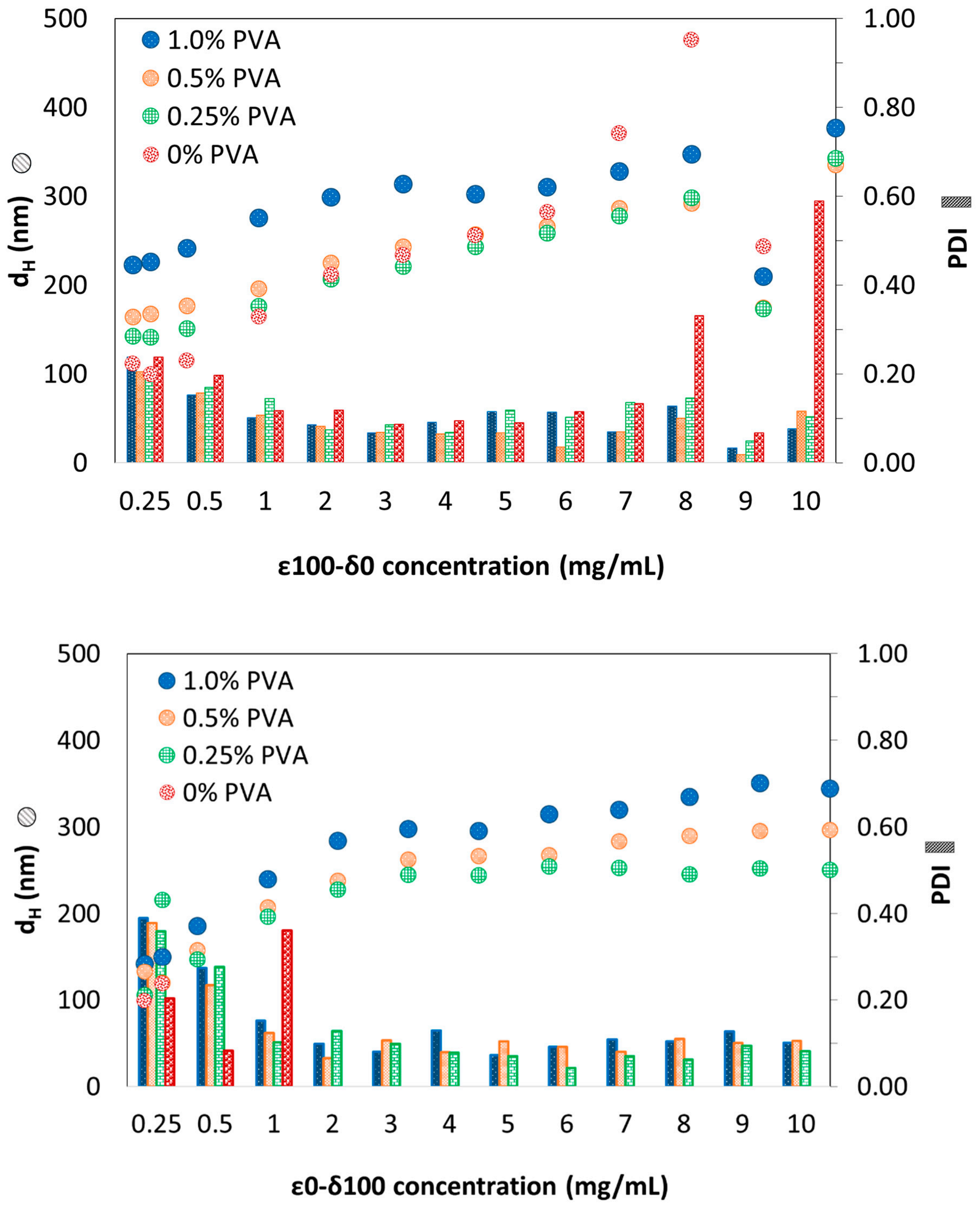


**Figure S1.** Hydrodynamic diameter (intensity-weighted distribution, circles) and PDI (bars) of the homopolymer ε100-δ0 and ε0-δ100 NPs over a range of PVA concentration used in the formulation.

# Supporting information

**Table S2.** Properties of PCL[BRP-187] NPs formulated from THF utilizing polymer concentration of 5 mg mL$^{-1}$ (n = 1 batch).

| εCL/δCL (mol %) | Purified NPs[a] | | Lyophilized NPs[b] | LC[c] | PVA[d] | Yield[e] |
|---|---|---|---|---|---|---|
| | $d_H$ (nm) | PDI | $d_H$ (nm) | PDI | (%, w/w) | (%) |
| **ε100-δ0** | 593 | 0.52 | 461 | 0.40 | 2.00 | 97 |
| **ε87-δ13** | 427 | 0.23 | 349 | 0.30 | 2.01 | 86 |
| **ε81-δ19** | 445 | 0.24 | 465 | 0.33 | 1.82 | 99 |
| **ε75-δ25** | 421 | 0.28 | 651 | 0.62 | 1.88 | 95 |
| **ε61-δ39** | 407 | 0.19 | 443 | 0.40 | 1.63 | 76 |
| **ε45-δ55** | 397 | 0.26 | 414 | 0.36 | 2.26 | 60 |
| **ε0- δ100** | 403 | 0.49 | 436 | 0.49 | 1.66 | 53 |

$d_H$ represents the intensity-weighted distribution.
(a) NPs measured after purification.
(b) NPs measured after lyophilization and subsequent resuspension in water.
(c) Determined by UV-VIS spectroscopy at $\lambda$ = 316 nm (n = 4) and calculated using LC = (mass of drug recovered) / (mass of particle recovered) x 100.
(d) The determination of PVA in the NPs (%, w/w) was performed according to the published protocol.[15](e) Yield = (mass of NPs recovered including PVA residue) / (mass of polymer + mass of drug) in the formulation x 100.

**Table S3.** Particle properties of empty PCL NPs prepared in THF with c = 2.5 mg mL$^{-1}$ (n = 2 batches) obtained by DLS and ELS measurements after purification and after lyophilization and subsequent resuspension (n = 2 for purified NPs, n = 1 for lyophilized NPs).

| εCL/δCL (mol %) | Purified NPs [a] | | Lyophilized NPs [b] | | ZP [b] | PVA [c] | Yield [d] |
|---|---|---|---|---|---|---|---|
| | $d_H$ (nm) | PDI | $d_H$ (nm) | PDI | (mV) | (%, w/w) | (%) |
| **ε100-δ0** | 250 | 0.08 | 250 | 0.21 | -33 | 2.0 | 93 |
| **ε87-δ13** | 177 | 0.05 | 253 | 0.27 | -23 | 2.0 | 68 |
| **ε81-δ19** | 193 | 0.06 | 268 | 0.28 | -38 | 1.8 | 70 |
| **ε75-δ25** | 196 | 0.05 | 279 | 0.33 | -32 | 1.9 | 76 |
| **ε61-δ39** | 170 | 0.08 | 220 | 0.21 | -13 | 1.6 | 54 |
| **ε45-δ55** | 163 | 0.06 | 205 | 0.23 | -33 | 2.3 | 51 |
| **ε0- δ100** | 212 | 0.08 | 285 | 0.30 | -40 | 1.7 | 67 |

$d_H$ represents the intensity-weighted distribution (five measurements) and zeta-potential (ZP) (three measurements).
(a) NPs measured after purification.
(b) NPs measured after lyophilization and subsequent resuspension in water.
(c) The determination of PVA in the NPs (%, w/w) was performed according to the published protocol.[15]
(d) Yield = (mass of NPs recovered – mass of found PVA) / (mass of polymer + mass of drug) in the formulation x 100.

# Supporting information

**Table S4.** DLS intensity-weighted size distribution of PCL[BRP-187] NPs of one formulation round after purification, as well as after lyophilization and resuspension in water.

<table>
<tr><th>εCL/ δCL</th><th>After purification</th><th>After lyophilization & resuspension</th></tr>
<tr><td>ε100-δ0</td><td>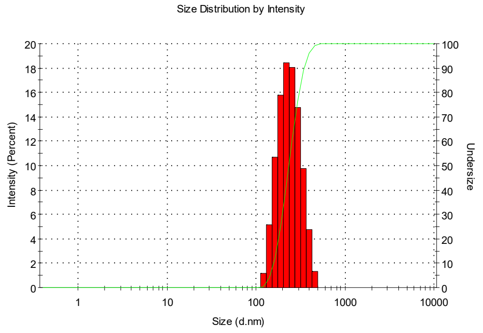
</td><td>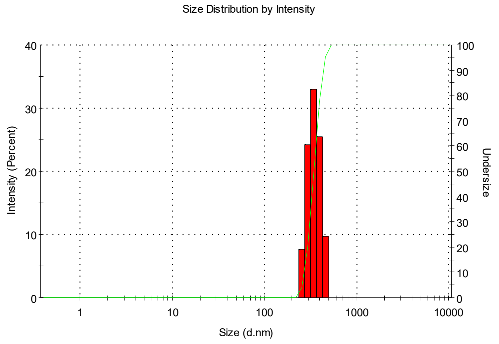
</td></tr>
<tr><td>ε87-δ13</td><td>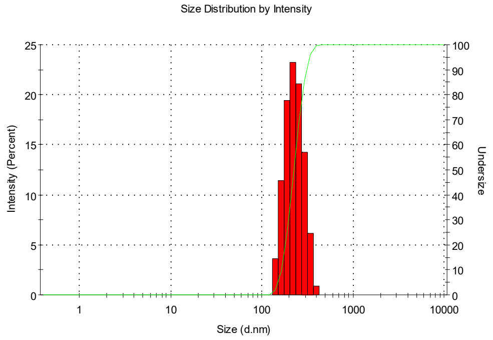
</td><td>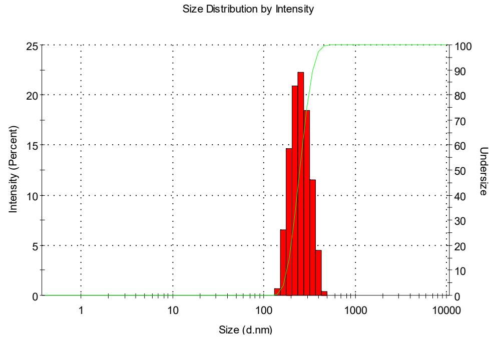
</td></tr>
<tr><td>ε81- δ19</td><td>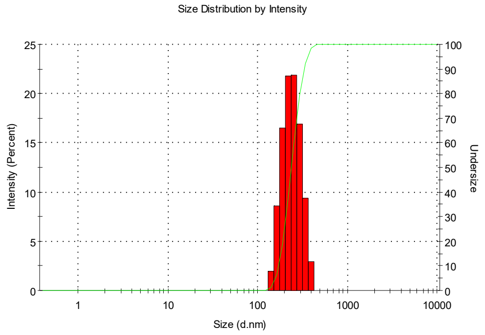
</td><td>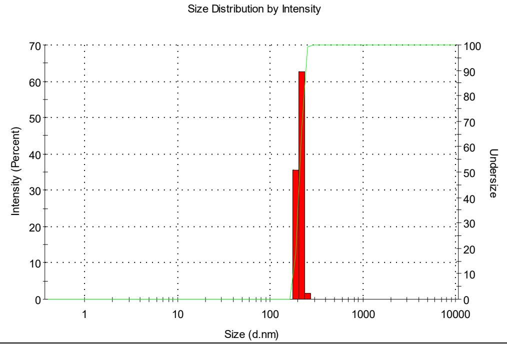
</td></tr>
<tr><td>ε75- δ25</td><td>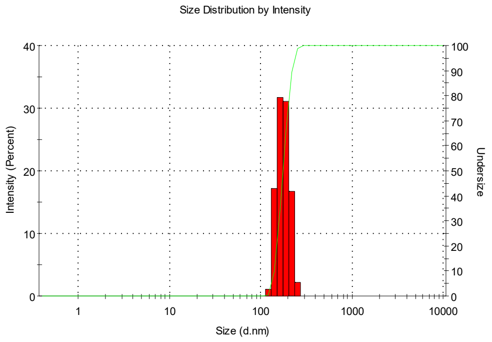
</td><td>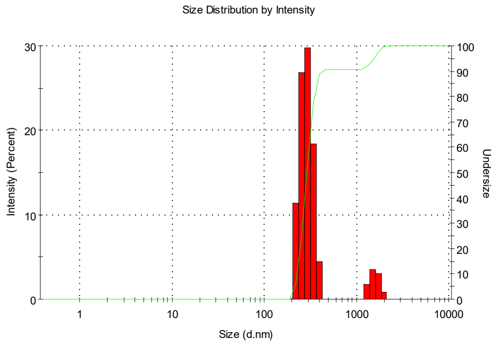
</td></tr>
</table>

# Supporting information

| | | |
|---|---|---|
| **ε61- δ39** | 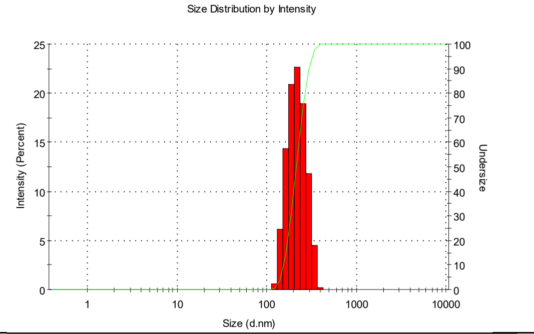 | 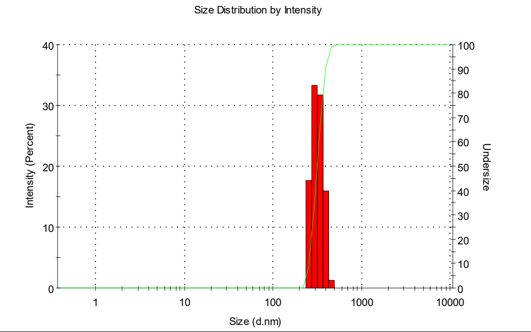 |
| **ε45- δ55** | 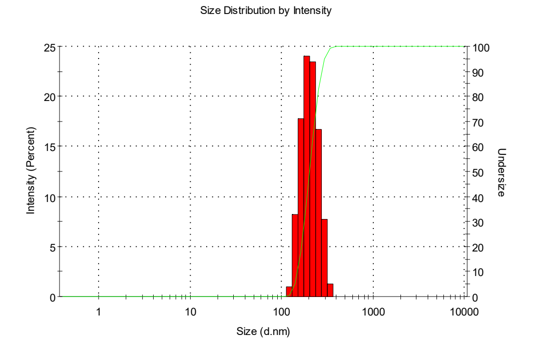 | 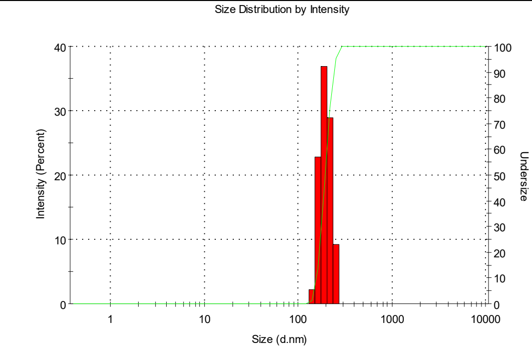 |
| **ε0- δ100** | 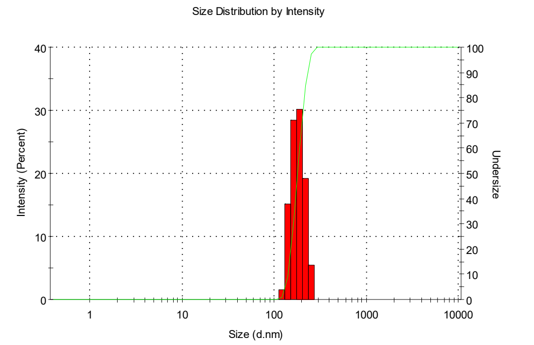 | 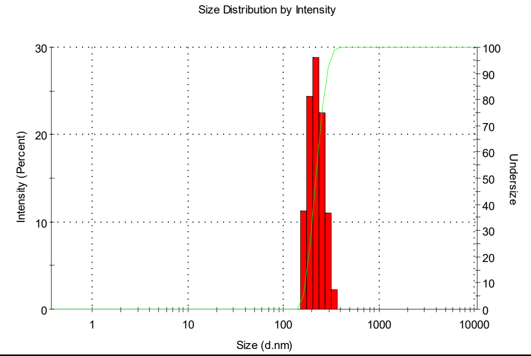 |

# Supporting information

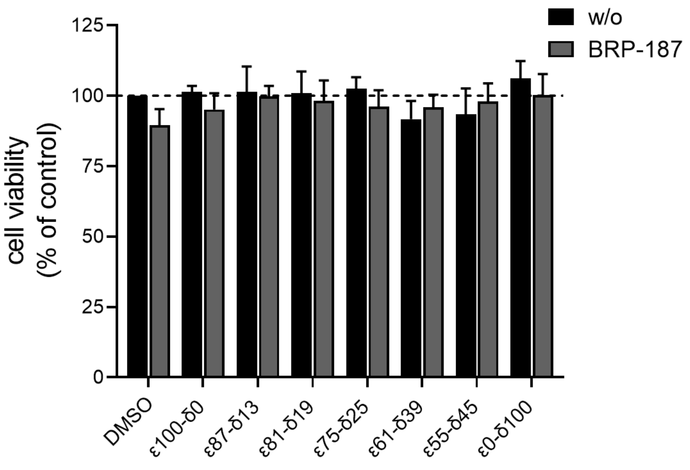


**Figure S2.** Cell viability measured with a Beckman ViCell XR cell counter by trypan blue staining. A total of 1 x 107 PMNL were diluted in PBS plus 0.1% of glucose and incubated with DMSO, BRP-187 (10 μM), empty PCL particles (labeled as w/o) or PCL particles with BRP-187 (labeled with BRP-187; respective amount to 10 μM BRP-187) for 5 h at 37 °C. Values are given as 5-LO products as a percentage of control (DMSO) (n = 3).

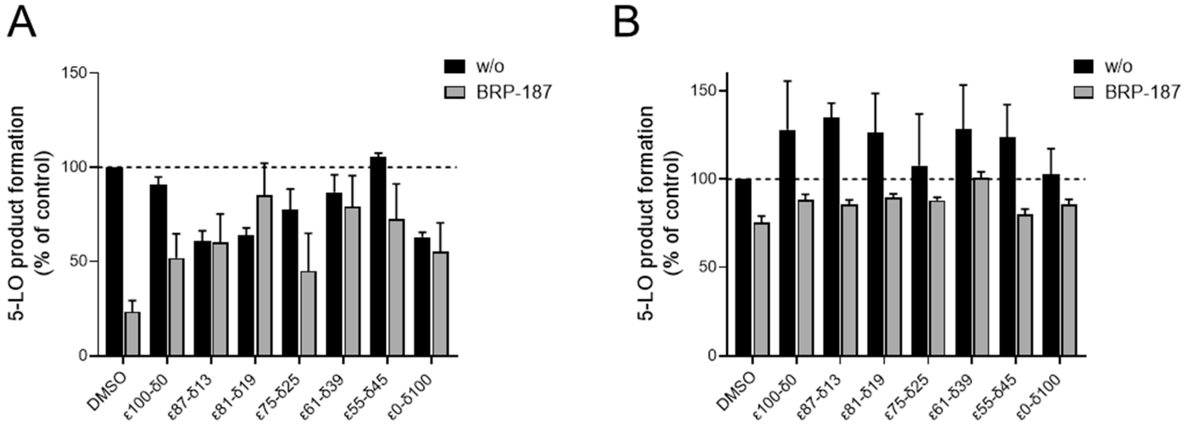


**Figure S3.** Measurement of 5-LO product formation as indicator for the inhibition of the drug target 5-lipoxygenase-activating protein (FLAP) by BRP-187.[37] A total of 5 x 106 polymorphonuclear leukocytes (PMNL) diluted in PBS containing 0.1% glucose and 1mM $CaCl_2$ were preincubated with DMSO, BRP-187 (0.3 μM), empty PCL particles (labeled as w/o) or PCL particles with BRP-187 (labeled as BRP-187; 0.3 μM respective BRP-187) for 1 h (A) and 2 h (B) at 37 °C and further stimulated with 2.5 μM A23187 for 10 min. The reaction was stopped with 1 mL ice-cold methanol containing 200 ng $mL^{-1}$ PGB1 as internal standard. Lipid mediators were extracted via solid-phase extraction (SPE) and analyzed with HPLC. Values are given as 5-LO products ($LTB_4$, its trans-isomers $_4$ and 5-HETE) as a percentage of control (DMSO) (n = 3).

# Supporting information

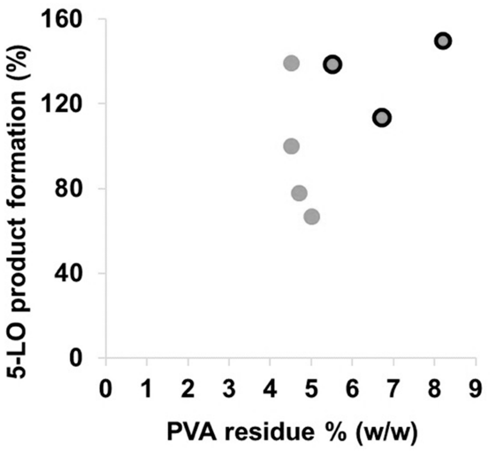


**Figure S4.** Influence of the residual PVA on the efficiency of drug-loaded PCL NPs on 5-LO inhibition. Black-circled data points represent PCL polymers with bulk degree of crystallinity below 10% and glass transition temperature $T_g < 37$ °C.
